# Chronic obstructive pulmonary disease upper airway microbiome is associated with select clinical characteristics

**DOI:** 10.1371/journal.pone.0219962

**Published:** 2019-07-23

**Authors:** Alexa A. Pragman, Katherine A. Knutson, Trevor J. Gould, Shane W. Hodgson, Richard E. Isaacson, Cavan S. Reilly, Chris H. Wendt

**Affiliations:** 1 Department of Medicine, Minneapolis Veterans Affairs Medical Center and University of Minnesota, Minneapolis, Minnesota, United States of America; 2 Division of Biostatistics, University of Minnesota, Minneapolis, Minnesota, United States of America; 3 University of Minnesota Informatics Institute, University of Minnesota, St. Paul, Minnesota, United States of America; 4 Research Service, Minneapolis Veterans Affairs Medical Center, Minneapolis, Minnesota, United States of America; 5 Department of Veterinary and Biomedical Sciences, University of Minnesota, St. Paul, Minnesota, United States of America; National and Kapodistrian University of Athens, GREECE

## Abstract

**Background:**

Chronic obstructive pulmonary disease (COPD) is an inflammatory lung disorder associated with lung microbiome dysbiosis. Although the upper airway microbiome is the source of the lung microbiome, the relationships between the oral, nasal, and sputum microbiota are incompletely understood. Our objective was to determine features that differentiate the oral, nasal, and sputum microbiome among subjects with stable COPD.

**Methods:**

We recruited 15 current or former smokers to provide oral and sputum samples on day 1. On day 2, another oral sample and a nasal sample were obtained. Each sample and control underwent DNA extraction, 16S V4 rRNA amplification, 16S V4 sequencing, and qPCR of 16S rRNA. Data were analyzed using dada2 and R.

**Results:**

Most (14 of 15) subjects were male with a mean age of 65.2. One subject had no pulmonary obstruction, while 5 had mild COPD, 7 had moderate COPD, and 2 had severe COPD. Three subjects (20%) were current tobacco users and 2 subjects (13%) used inhaled corticosteroids (ICS). Subjects had a mean of 49.1 pack-years of tobacco exposure. Bacterial biomass was associated with anatomic site, but no differences in biomass were observed with age, FEV1 percent predicted (FEV1pp), ICS use, smoking status, or edentulous state. Shannon index was associated with site (lower nasal diversity than oral and sputum diversity, *p*<0.001), but not age, ICS use, FEV1pp, tobacco use, or edentulous state. β-diversity was illustrated by principal coordinate analysis using Bray-Curtis dissimilarity and PERMANOVA analyses, showing sample clustering by anatomic site (*p* = 0.001) with nasal samples forming a cluster separate from the combined oral wash samples and sputum samples. Clustering was also observed with ICS use (*p* = 0.029) and edentulous state (*p* = 0.019), while FEV1pp and current tobacco use were not significant. In an amplicon sequencing variant (ASV)-level analysis of oral samples using a linear regression model with Benjamini-Hochberg correction at an FDR<0.10, 10 ASVs were associated with age while no ASVs were associated with FEV1pp or smoking status. Sputum sample analysis demonstrated that 51 ASVs (25 unique genera) were associated with age, 61 ASVs (32 genera) were associated with FEV1pp, and no ASVs were associated with smoking status. In a combined dataset, the frequent exacerbator phenotype, rather than ICS use, was associated with decreased sputum Shannon diversity.

**Conclusions:**

Among the upper airway microbiota of COPD subjects, anatomic site was associated with bacterial biomass, Shannon diversity, and β-diversity. ICS use and edentulous state were both associated with β-diversity. Age was associated with taxa relative abundance in oral and sputum samples, while FEV1pp was associated with taxa relative abundance in sputum samples only.

## Introduction

Chronic obstructive pulmonary disease (COPD) is a progressive, inflammatory lung disease that is most commonly caused by tobacco exposure. COPD exacerbations are episodes of worsening lung symptoms and are associated with excess morbidity and mortality. Half of all COPD exacerbations are associated with bacterial lung infection, most commonly with organisms such as *Haemophilus influenzae*, *Moraxella catarrhalis*, and *Streptococcus pneumoniae* [1]. These and other bacteria often persist in the lung microbiota of COPD patients. The persistence of these organisms—even during periods of clinical stability—is associated with increased lung symptoms and inflammation [2].

Chronic inflammatory lung diseases such as COPD are associated with changes in the lung microbiota (often termed dysbiosis). We and others have described the COPD-associated lung microbiota as consisting primarily of *Streptococcus*, *Veillonella*, *Prevotella*, *and Haemophilus*, and *Rothia* [3–13]. Due to significant taxa overlap between the oral, nasal, and lung microbiota, it appears that aspiration of upper airway taxa is the source of the lung microbiota [8,14–16]. The relationships between the upper airway microbiota and the lung microbiota as well as factors that influence these microbiota are areas of active research.

Despite this progress, we still lack a complete understanding of upper airway and lung microbiota changes associated with several important clinical factors related to COPD. For instance, older age, declining lung function, tobacco exposure, tooth loss, and use of medications such as inhaled corticosteroids (ICS) are common among patients with COPD. Currently, we lack robust information on associations between these clinical factors and the lung and upper airway microbiota. Studies to examine these associations are complicated by the fact that many of these clinical factors are closely associated with each other and COPD progression.

The effects of aging on the upper airway and lung microbiota are not well understood. We previously evaluated the COPD lung microbiota and observed that aging was more closely associated with changes in alpha diversity than was obstruction severity [5,17]. Several groups have established an association between decreased lung microbiota alpha diversity and worsening FEV1 percent predicted (FEV1pp) [3,18]. Tobacco use has been associated with decreased oral microbiota richness [19], increased alpha diversity of the esophagus [20] and lung tissue [21], and altered taxa abundance of the oral microbiota [22]. Tobacco exposure has not been associated with significant changes in sputum taxa abundance or alpha diversity [19,22].

Associations between tooth loss and changes in the oral microbiota have been studied in the general population, but not in the setting of COPD or tobacco use. Decreased Shannon diversity was correlated with edentulous state and complete (as opposed to partial) denture use. Dental plaque has higher alpha diversity than the denture-associated microbiota [23].

The use of ICS and associated oral and sputum microbiota changes have not been definitively addressed. In an earlier study, we noted shifts in beta diversity that coincided with ICS use [5]. In an interventional study, ICS use was associated with increased sputum bacterial load. Multiplex PCR also suggested that a small number of select COPD-associated taxa increased in abundance with ICS use. Clinically, ICS use is associated with a decreased rate of COPD exacerbations and an increased risk of pneumonia [24].

Therefore, we undertook the present study to determine if clinical factors (i.e., aging, declining lung function, tobacco use, tooth loss, and ICS use) are associated with features of the oral, nasal, and sputum microbiome among subjects with stable COPD. Our central hypothesis is that many of the upper airway and lung microbiota changes currently associated with COPD may also be associated with other co-occurring clinical factors. We hope that by identifying these associations we may enhance our understanding of the COPD lung microbiota and identify potentially-modifiable factors that may improve the lung microbiota and COPD therapies.

## Materials and methods

### Subjects

Fifteen subjects pre-operatively admitted to the Minneapolis VA Medical Center for lung lobectomy (for suspected or confirmed lung cancer) and who were over the age of 40 were recruited at the Minneapolis VA Medical Center between February 2, 2016 and January 3, 2017. Patients were excluded if they had used antibiotics or systemic corticosteroids in the last 1 month or had asthma. Neither the results of pulmonary function testing nor a clinical diagnosis of COPD were included among the inclusion or exclusion criteria. The study was approved by the Minneapolis VA Medical Center Institutional Review Board (#4348-B); written informed consent was obtained from all participants.

### Sample acquisition

Subjects provided oral and sputum samples on day 1; another oral sample and a nasal sample were obtained the morning of day 2, after fasting overnight. Oral samples were obtained by having subjects swish sterile water in their mouths for 30 seconds and then expectorating into a DNA-free tube. After oral wash sampling, sputum was obtained by spontaneous expectoration (if the subject was able to do so) or by induction using 3% saline (0.9% saline if FEV_1_ <40% predicted) and expectorating into a DNA-free container. Nasal samples were obtained by swabbing each anterior nare/nasopharynx with a nylon-flocked swab (Copan Diagnostics, Inc., Murrieta, CA), which were combined in a sterile, DNA-free tube. One subject was unable to provide a sputum sample. Oral washes and sputa were weighed and all samples were immediately frozen at -80°C until sample processing. DNA contamination of reagents and equipment was evaluated using both liquid protocol and swab protocol extraction controls, which were processed and analyzed alongside the experimental samples.

### DNA extraction and 16S rRNA gene sequencing

After thawing, sputum was treated with sputolysin and incubated at 37°C for 15 minutes prior to pelleting and resuspension in MO BIO PowerSoil DNA Isolation Kit buffer (QIAGEN, Germantown, MD). Oral wash and liquid protocol negative control samples were thawed, pelleted, and resuspended in power bead tube buffer solution (QIAGEN). Nasal swabs and swab protocol controls were placed in the power bead tube and vortexed. Once resuspended, all sample types underwent DNA isolation according to the MO BIO PowerSoil DNA Isolation Kit protocol. 16S rRNA V4 amplicons were generated via 20 PCR amplification cycles using primers 515F and 806R. Amplicons were sequenced with an Illumina MiSeq 600 cycle v3 kit using paired-end reads at the University of Minnesota Genomics Center.

### Quantitative PCR (qPCR)

To determine 16S rRNA gene copy numbers, qPCR was performed in triplicate for all samples and controls. Twenty μl reactions using 16S rRNA qPCR primers 338-F (5’-ACTCCTACGGGAGGCAGCAG-3’) and 518-R (5’-ATTACCGCGGCTGCTGG-3’) at a final concentration of 0.67 μM for each primer. The LightCycler 480 SYBR Green I Master Kit (Roche) was utilized for qPCR on the Roche LightCycler 96. Cycling conditions were 50°C for 2 min, 95°C for 2 min, then 40 cycles of 95°C for 15 sec, 58°C for 15 sec, and 72°C for 30 sec, followed by a melting curve. The standard curves for absolute quantification of 16S rRNA gene copy numbers were constructed using the DH5α *E*. *coli* strain by initially creating an end-point PCR product of the DH5α strain with universal 16S rRNA gene primers Bact-27F and Bact-1492R [25]. The standard curve was created using ten-fold serial dilutions of the *E*. *coli* PCR product. Oral wash and sputum sample copy numbers were normalized to sample mass.

### Data processing

**Fig 1:**
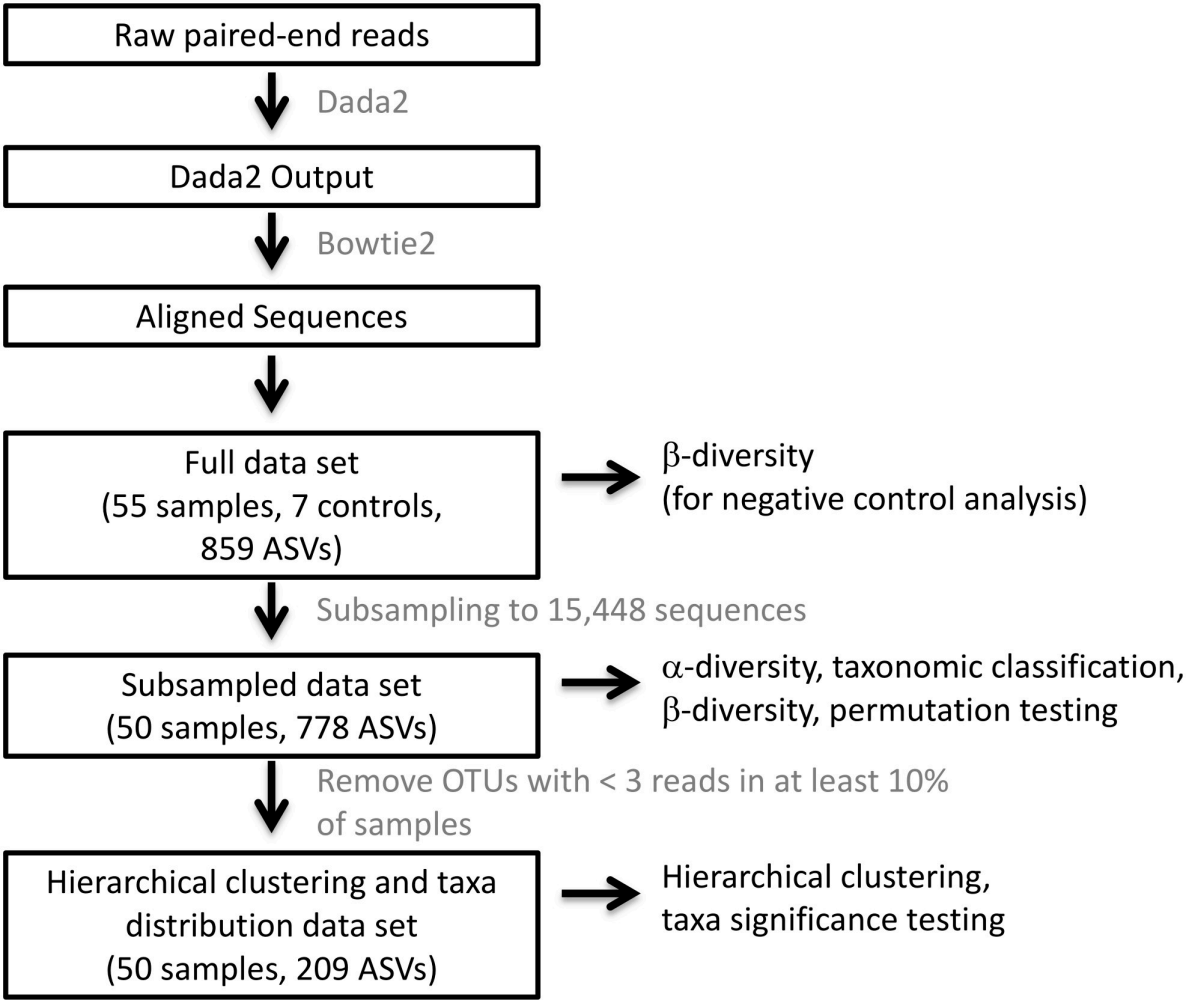
Data processing flow chart. DNA sequences were processed into data sets using the software tools and procedures as described in the text and illustrated here as a pipeline. Boxed black text indicates significant steps in the pipeline or data sets produced for specific analyses. Gray text specifies the software tools or procedures used in the pipeline. Unboxed black text specifies the analyses performed on the indicated data sets.

For both the present study and the exacerbation phenotype study, Dada2 was used to filter, trim, dereplicate, merge paired reads, and remove primers, phix, and bimeras [26]. Bowtie2 [27] was used to remove human sequences prior to aligning sequences using the Ribosomal Database Project (RDP) Classifier [28] with the SILVA database [29]. The full data set underwent β-diversity analysis with Bray-Curtis dissimilarity to visualize control and sample similarities and determine subsampling depth. Subsampling the dataset from the present study to 15,448 sequences eliminated all negative control samples and 5 nasal samples. This subsampled data set, which consisted of 778 amplicon sequencing variants (ASVs), was used in subsequent analyses. Subsampling the dataset from the exacerbation phenotype study to 25,955 sequences eliminated all negative control samples and one FE sputum sample. This subsampled data set, which consisted of 398 amplicon sequencing variants (ASVs), was used in subsequent analyses. Alpha diversity indices (Shannon and Simpson indices) were calculated using vegan while β-diversity analyses utilizing the Bray-Curtis dissimilarity were performed using phyloseq. Prior to hierarchical clustering and taxa distribution analyses, additional filtering was performed to remove ASVs that did not have at least 3 reads in 10% of all samples. 209 ASVs remained in this data set. A data processing flow chart is provided in Fig 1.

### Statistical analysis

Statistical power was estimated using the simulation-based power calculation for a mixed model implemented using the package simr v.1.0.5 in R. To compare 16S rRNA copy numbers, a generalized estimating equation with an exchangeable correlation structure was used with anatomic site as a predictor and patient as a random effect. Pairwise *t*-tests with Holm correction were used to compare 16S copies between anatomic sites. Additional clinical factors were added to the model individually. Alpha diversity indices (Shannon and Simpson diversity) were evaluated by site using linear mixed models with diversity as a response, site as predictor, and patient as a random effect. Paired *t*-tests with Benjamini-Hochberg adjustment were used to compare sites. Permutation tests with estimates from linear models were performed to evaluate clinical predictors. Alpha diversity was the response, with site, age, FEV1pp, current tobacco use, and edentulous state as predictors. Oral wash samples were combined and *p*-values were FDR adjusted for the number of covariates. Hierarchical clustering was performed using the hclust function and Euclidean distance metric after filtering the dataset as described above. β-diversity analyses were conducted using Bray-Curtis dissimilarity and PERMANOVA analyses with default parameters. Mean within- and between-subject similarity was compared using permutation tests for the 6 possible comparisons between the 4 sample sites. The mean Bray-Curtis within-subject dissimilarity for each of the 6 comparisons was calculated. To calculate *p*-values for within- vs. between-subject comparisons, we used permutation tests to estimate the mean between-subject dissimilarity for each pair of samples. Similarly, to compare oral samples with the sputum samples, we calculated the mean within-subject Bray-Curtis dissimilarities for sputum samples and oral wash 1 as well as sputum samples and oral wash 2. The Wilcoxon signed rank permutation test was used to test the null hypothesis of no significant difference between sputum-oral wash 1 similarity and sputum-oral wash 2 similarity within patients. Clinical factors associated with ASV abundance were determined separately for oral wash and sputum samples. To determine sputum ASVs associated with clinical factors, we fit a linear regression model with log(reads+1) as the response and age, FEV1pp, and smoking status as explanatory variables. To determine oral wash ASVs associated with clinical factors, we fit linear mixed effects regression models with patient as random effect, log(reads+1) as the response, and age, FEV1pp, and smoking status as explanatory variables. For both sites, each clinical variable was fit individually and *p*-values were adjusted using Benjamini-Hochberg. After combining the present study dataset with our exacerbation phenotype dataset, we used a linear mixed model with alpha diversity (Shannon index) as a response, patient as a random effect and phenotype and clinical factors as fixed effects. Clinical factors were evaluated individually unless otherwise stated and *p*-values were determined with permutation tests. All permutation tests employed 1,000 permutations. All analyses were performed in R version 3.4.2.

## Results

### Characteristics of the study participants

We estimated 15 subjects were necessary to have 80% power to detect a difference of 0.75 in Shannon diversity between anatomic sites. Our observed effect size between nasal sample Shannon diversity and oral wash or sputum Shannon diversity was >1.5, indicating that we were adequately powered to detect a difference in alpha diversity between these sites. Most subjects were male with a mean age of 65.2 years. Most subjects had mild or moderate COPD by lung function criteria. One subject did not have COPD and two subjects had severe COPD. Most were former tobacco users (Table 1).

**Table 1:** Demographic and clinical characteristics of study participants.

	Descriptives
n	15
Age, mean (sd)	65.20 (4.51)
Gender, Male (%)	14 (93.3)
BMI, mean (sd)	27.65 (5.98)
Race, Caucasian/White (%)	14 (93.3)
COPD Severity, n (%)^a^	
Mild [FEV1pp ≥ 80%]	5 (33.3)^a^
Moderate [FEV1pp = 50–79%]	7 (46.6)
Severe [FEV1pp 30–49%]	2 (13.3)
ICS Use, Yes (%)	2 (13.3)
Pack years of smoking, mean (sd)	49.13 (19.6)
Current tobacco use, Yes (%)	3 (20.0)
Diabetes, Yes (%)	1 (6.7)
Edentulous, n (%)	3 (20.0)
Current alcohol use, Yes (%)	9 (60.0)
Gastroesophageal reflux disease, Yes (%)	7 (46.7)
Proton pump inhibitor use, Yes (%)	4 (26.7)
Oral steroid or antibiotic use in last 1 month, Yes (%)	0 (0)
Last dental visit, less than or equal to 6 months (%)	8 (53.3)
Lung cancer, n (%)	14 (93.3)
Adenocarcinoma, n (%)^b^	6 (40.0)^b^
Squamous Cell Carcinoma, n (%)^b^	8 (53.3)^b^
Large Cell Neuroendocrine tumor, n (%)	1 (6.7)
Unknown, n (%)^c^	1 (6.7)^c^

^a^One subject did not have COPD by lung function criteria.

^b^Subject 12 had 3 lung tumors (2 adenocarcinomas, 1 squamous cell carcinoma), which are included here individually; 16 independent lung tumors were diagnosed in 14 subjects.

^c^One surgery was cancelled for clinical reasons and no tissue diagnosis was made.

### 16S rRNA copy number is associated with site, but not clinical factors

**Fig 2:**
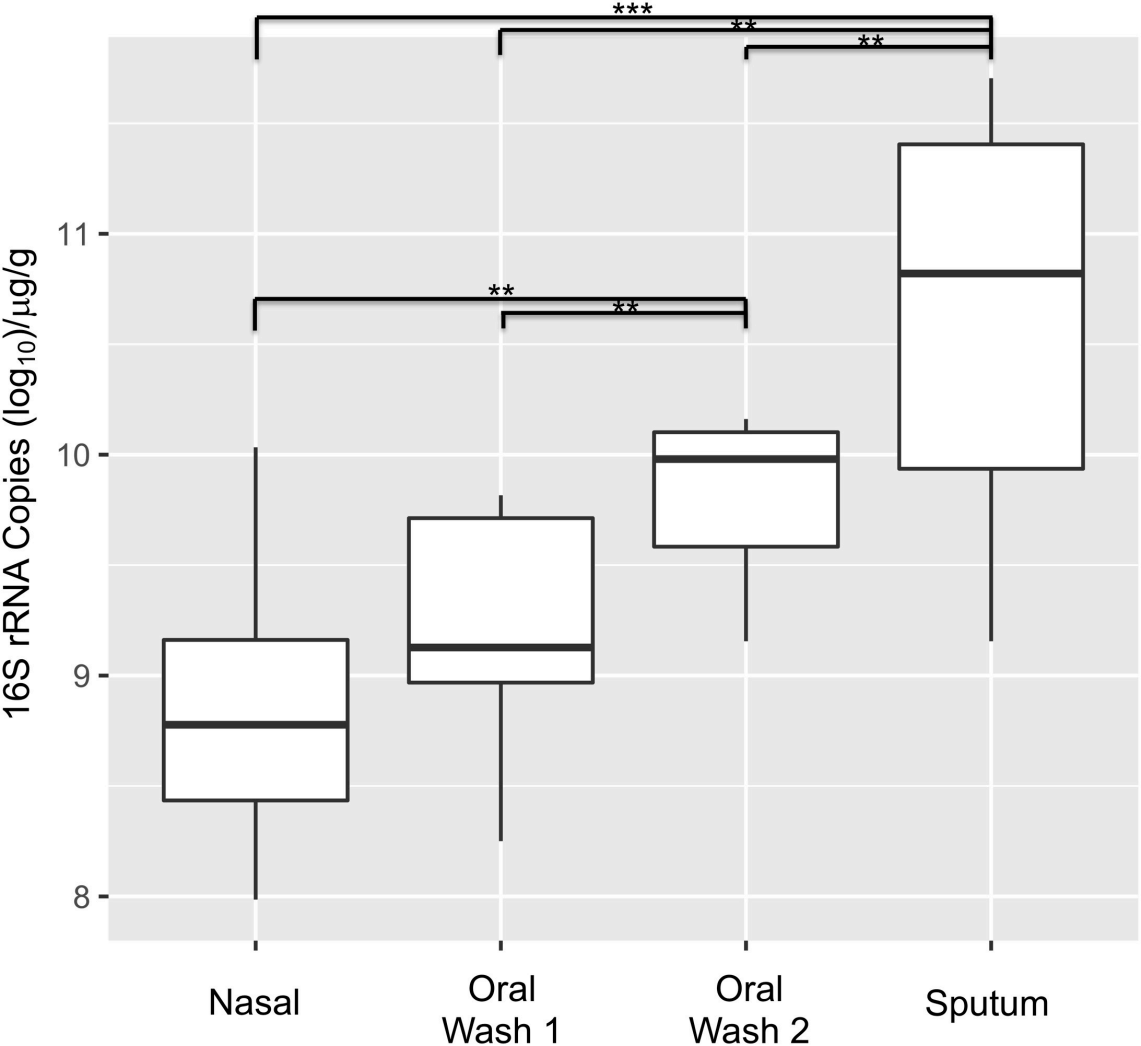
16S rRNA copy number is associated with site. A generalized estimating equation model with an exchangeable correlation structure was used with anatomic site as a predictor and patient as a random effect. Anatomic site was a significant predictor of 16S copy numbers (*p*<0.001). Follow-up pairwise *t*-tests revealed that all pairwise comparisons were significant with the exception of the nasal-oral wash 1 comparison (*p* = 0.08). Inclusion of smoking status, edentulous state, age, and FEV1pp in the model (in addition to anatomic site) resulted in non-significant *p*-values for the above-mentioned clinical factors. (***p<0.0001; **p = 0.001–0.0001).

16S rRNA copy numbers were determined for each subject and control sample and normalized to sample mass. Sputum samples contained the greatest number of 16S rRNA copies (mean 5.18 x 10^10^/μl/g), followed by oral wash 2 (OW2 mean 6.67 x 10^9^/μl/g), oral wash 1 (OW1 mean 1.52 x 10^9^/μl/g), nasal samples (mean 7.13 x 10^8^/μl/g) and negative control samples (mean 4.92 x 10^5^/μl/g, not shown; *p* <0.001). *Post-hoc* pairwise testing for all anatomic sites indicated significant differences across all pair-wise comparisons except the OW1-nasal comparison. Although anatomic site was a significant predictor of 16S copy numbers, the independent addition of multiple clinical factors (including current smoking status, edentulous state, age, and FEV1pp) resulted in non-significant *p*-values for all clinical factors (Fig 2). OW 1, which was obtained mid-day after a 2-hour fast, contained significantly fewer 16S rRNA copies than OW2, which was obtained in the early morning after an overnight fast.

### Samples are distinct from negative controls

Control samples contained 107–3,128 reads (mean 768); nasal samples contained 16–53,762 reads (mean 18,630); OW1 samples contained 25,657–70,780 reads (mean 44,378); OW2 samples contained 34,311–75,633 reads (mean 52,277); and sputum samples contained 34,760–95,538 reads (mean 55,326). The full data set underwent β-diversity analysis with Bray-Curtis dissimilarity to visualize control and sample similarities and determine subsampling depth (not shown). Subsampling to 15,448 reads eliminated all negative control samples and five nasal samples. Preliminary analyses on the subsampled dataset indicated that although the two oral wash samples contained significantly different 16S rRNA copy numbers, they were not significantly different from one another on either α- or β-diversity analyses. Therefore, unless otherwise noted, in subsequent analyses OW1 and OW2 samples are pooled together.

### Alpha diversity is associated with site

**Fig 3:**
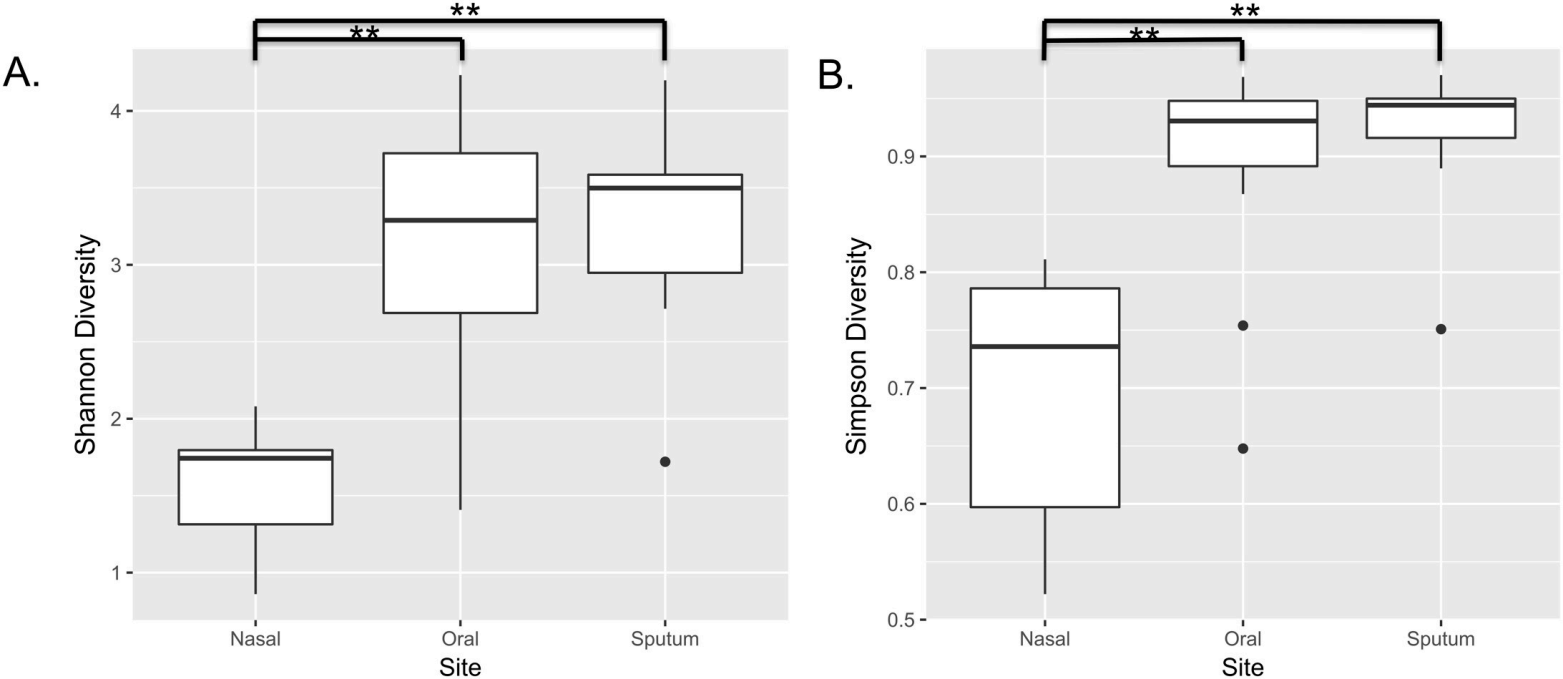
Alpha diversity is associated with site. Shannon and Simpson diversity were determined and the results were evaluated by site. There were 9 nasal samples, 28 oral samples (combined oral wash 1 and 2) and 13 sputum samples from 15 subjects. A) Nasal samples were least diverse, with a mean Shannon index of 1.57, followed by oral samples with a mean Shannon index of 3.19, and sputum samples with a mean Shannon index of 3.31. A linear mixed model including patient-level random effects revealed significant differences in Shannon diversity with respect to site (*p*<0.001). Paired *t*-tests with Benjamini-Hochberg adjustment were used to evaluate the relationship between sites, revealing significant differences between the nasal-oral sites and nasal-sputum sites (both *p*-values <0.001), while there was not a significant difference between sputum and oral wash sites (*p* = 0.91). B) Nasal samples were least diverse, with a mean Simpson index of 0.70, followed by oral samples with a mean Simpson index of 0.91, and sputum samples with a mean Simpson index of 0.92. A linear mixed model patient-level random effects revealed significant differences in Simpson diversity with respect to site (*p*<0.001). Paired *t*-tests with Benjamini-Hochberg adjustment were used to evaluate the relationship between sites, revealing significant differences between the nasal-oral sites and nasal-sputum sites (both *p*-values <0.001), while there was not a significant difference between sputum and oral wash sites (*p* = 0.67). To evaluate potential clinical predictors of alpha diversity, a permutation test with a general linear model was performed. Age, FEV1pp, current tobacco use, and edentulous state were individually included with site as predictors. *P*-values were FDR adjusted. For both Shannon and Simpson diversity models, only anatomic site was a significant predictor of alpha diversity.

Shannon and Simpson diversity indices, which incorporate both microbial richness and evenness, were determined for each subject sample. Alpha diversity indices were lowest for nasal samples (mean Shannon index 1.57), followed by oral wash samples (mean Shannon index 3.19) and then sputum (mean Shannon index 3.31). A linear mixed model revealed significant differences in Shannon diversity with respect to site (*p*<0.001). Paired *t*-tests with Benjamini-Hochberg adjustment were used to evaluate the relationship between sites, revealing significant differences between the nasal-oral sites and nasal-sputum sites (both *p*-values <0.001), while there was not a significant difference between sputum and oral wash sites (*p* = 0.91). Using Simpson diversity, nasal samples were least diverse, with a mean Simpson index of 0.70, followed by oral samples with a mean Simpson index of 0.91, and sputum samples with a mean Simpson index of 0.92. A linear mixed model revealed significant differences in Simpson diversity with respect to site (*p*<0.001). Paired *t*-tests with Benjamini-Hochberg adjustment were used to evaluate the relationship between sites, revealing significant differences between the nasal-oral sites and nasal-sputum sites (both *p*-values <0.001), while there was not a significant difference between sputum and oral wash sites (*p* = 0.67). We performed additional testing to evaluate for other clinical factors (age, FEV1pp, current tobacco use, and edentulous state) that may be associated with alpha diversity. For both Shannon and Simpson diversity models, only anatomic site was a significant predictor of alpha diversity (Fig 3).

**Fig 4:**
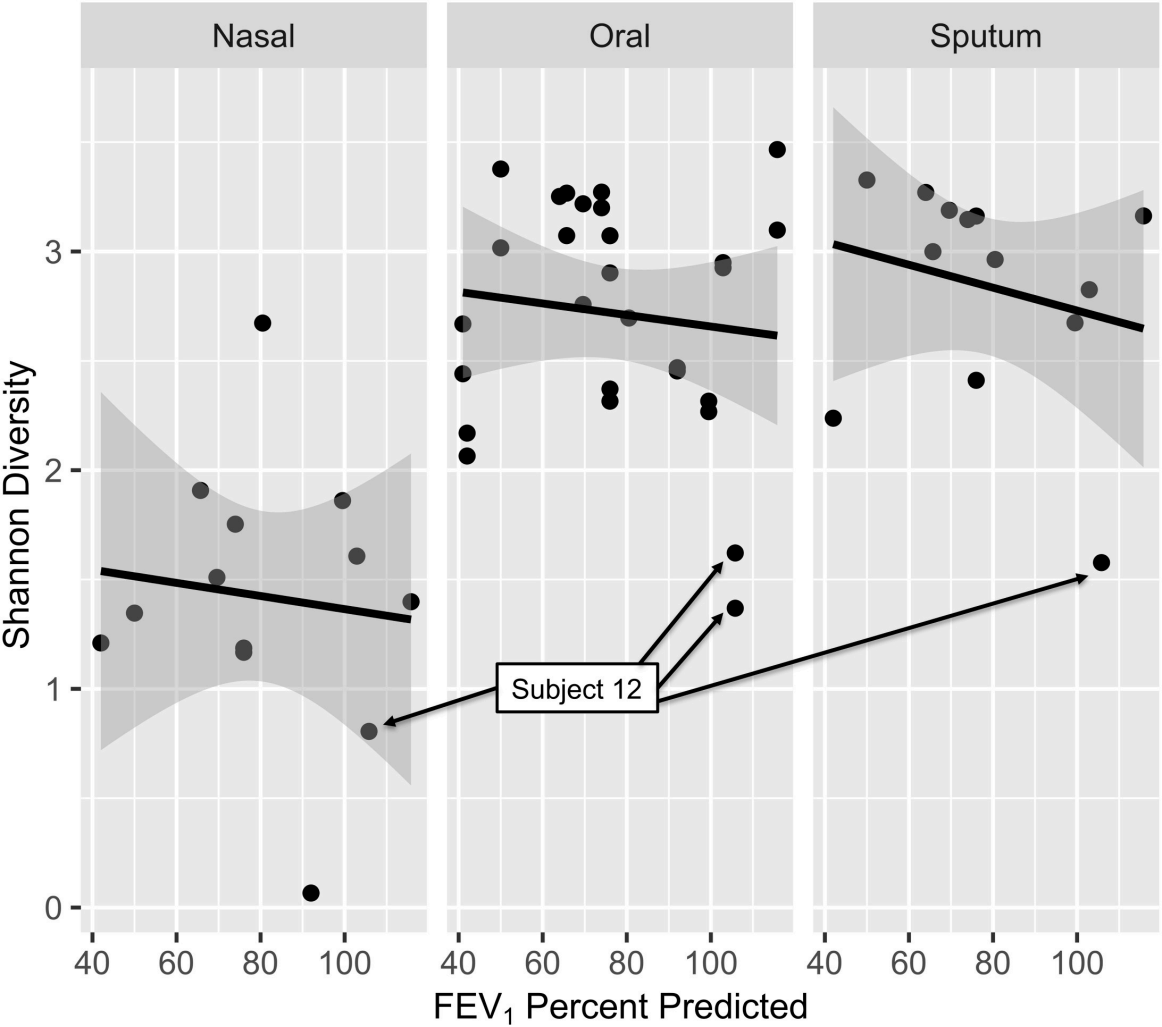
Low alpha diversity is consistently seen for one subject. Shannon diversity by anatomic site and FEV1pp is presented here with black lines illustrating predicted Shannon diversity at each site based on FEV1pp. Gray shading indicates the 95% confidence interval for the regression line. Although FEV1pp was not a significant predictor of alpha diversity in our dataset, we noted that all four samples from one subject (12, labeled above) exhibited very low diversity, particularly at the oral and sputum sites. This subject was unique in our dataset in that she was the only woman and had three independent lung tumors.

Although FEV1pp was not a significant predictor of alpha diversity in our dataset, we noted that all four samples from subject 12 exhibited very low diversity. This subject was unique in our dataset in that she was the only woman in the study and at the time of surgery she was found to have three independent lung tumors (Fig 4).

### Nasal samples are distinct from the combined oral wash and sputum samples

**Fig 5:**
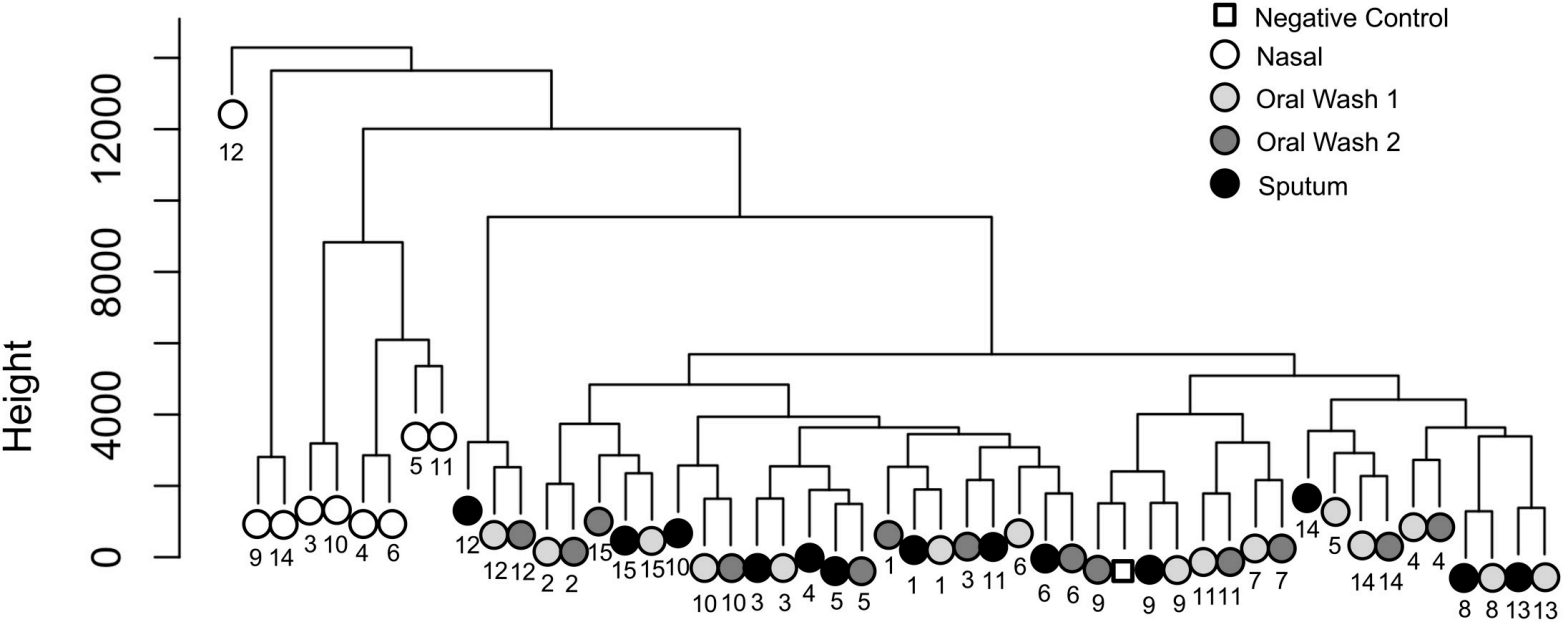
Nasal samples are distinct from the combined oral wash and sputum samples. Hierarchical clustering was performed using the hclust function after filtering the dataset as described in the text. Nasal samples cluster together at left, while oral wash samples and sputum samples are intermixed at right. In many cases, the two oral wash samples from the same subject are the nearest neighbors, although sputum samples are often closely associated with one of the oral wash samples from the same subject. Subjects are identified by number and sample type is indicated by color (see legend).

Hierarchical clustering was performed after filtering the dataset as described in the methods and Fig 1. Nasal samples cluster together at left, while oral wash samples and sputum samples are intermixed at right. In many cases, the two oral wash samples from the same subject are the nearest neighbors, although sputum samples are often closely associated with one of the oral wash samples from the same subject (Fig 5). All four samples from subject 12 again appear as outliers in this analysis.

### Beta diversity demonstrates clustering by anatomic site, inhaled corticosteroid use, and edentulous state

**Fig 6:**
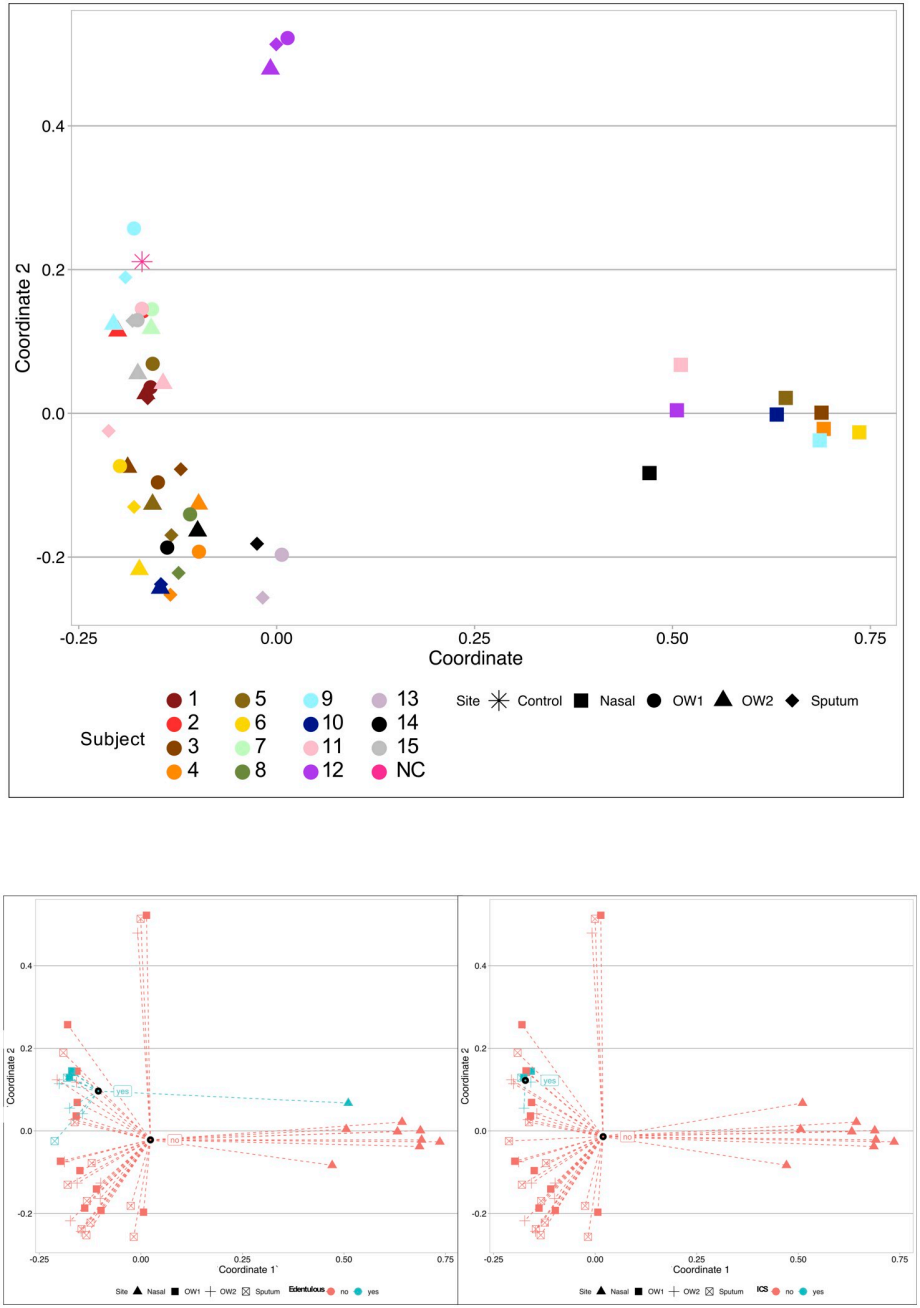
Beta diversity demonstrates clustering by anatomic site, inhaled corticosteroid use, and edentulous state. Principal coordinate analysis (PCoA) using Bray-Curtis dissimilarity followed by PERMANOVA analyses were used to identify clinical factors responsible for sample clustering on β-diversity analyses. **Upper panel**: Anatomic site is indicated by symbol, while each subject is labeled with a unique color. Anatomic site (nasal vs. sputum vs. oral wash 1 vs. oral wash 2) was again a significant predictor of beta-diversity (*p* = 0.001). Oral wash 1 and oral wash 2 samples did not form separate clusters (*p* = 0.93); sputum samples also failed to cluster separately from either of the oral wash sample groups (*p* = 0.62). This confirms that nasal samples (triangles at right) were responsible for the clustering by anatomic site. Neither FEV1pp (≥80% vs. <80%; *p* = 0.077) nor current tobacco use (current tobacco users or recent quitters vs. remote quitters; *p* = 0.062) were associated with β-diversity. Notably, the sputum and oral wash samples from subject 12 clustered together at the upper left, separate from the other subjects’ oral washes and sputa (these samples from subject 12 were also outliers on α-diversity analyses, see Fig 4). The nasal sample from this subject clustered with the other subjects’ nasal samples. **Lower Left**: Anatomic site is indicated by symbol, while color is used to indicate edentulous state. Samples from edentulous subjects clustered separately from dentate subjects (*p* = 0.019). **Lower Right**: Anatomic site is indicated by symbol, while color is used to indicate ICS use. ICS users’ samples clustered separately from non-ICS users’ samples (*p* = 0.029).

Principal coordinate analysis (PCoA) using Bray-Curtis dissimilarity followed by PERMANOVA analyses were used to identify clinical factors responsible for sample clustering on beta diversity analyses (Fig 6). Anatomic site (nasal vs. sputum vs. OW1 vs. OW2) was again a significant predictor of beta diversity (*p* = 0.001). OW1 and OW2 samples did not form separate clusters (*p* = 0.93); sputum samples also failed to cluster separately from either of the oral wash sample groups (*p* = 0.62). This confirms that nasal samples were responsible for the clustering by anatomic site. ICS users’ samples clustered separately from non-ICS users’ samples (*p* = 0.029), while samples from edentulous subjects clustered separately from dentate subjects (*p* = 0.019). Neither FEV1pp (≥ 80% vs. <80%; *p* = 0.077) nor current tobacco use (current tobacco users or recent quitters vs. remote quitters; *p* = 0.062) were associated with β-diversity, though there was some evidence for both associations. Notably, the sputum and oral wash samples from subject 12 clustered together at the upper left, separate from the other subjects’ oral washes and sputa. The nasal sample from this subject clustered with the other subjects’ nasal samples. Inspection of the taxa found in all 4 samples from subject 12 found that samples from this subject were enriched with specific *Veillonella*, *Granulicatella*, *Anaerococcus*, *Fusobacterium*, *Prevotella*, *Bacteroides*, *Acinetobacter*, *Staphylococcus*, and *Streptococcus* ASVs in comparison to samples from other subjects. The nasal sample from subject 12 was dominated by *Staphylococcus* and *Corynebacterium*, while the two oral wash samples were dominated by *Streptococcus* (3 ASVs), *Rothia*, and *Veillonella*. The sputum sample from subject 12 was dominated by *Streptococcus* (3 ASVs) and *Rothia* (2 ASVs).

### There is greater within-subject than between-subject similarity for all anatomic site comparisons

We compared the mean within-subject similarity with the mean between-subject similarity for all 6 possible comparisons between the 4 samples obtained (Table 2). We calculated the Bray-Curtis dissimilarities between each possible within-subject sample pair and obtained a mean within-subject dissimilarity for each pair of samples. We estimated the mean between-subject dissimilarity for each pair of samples. We tested the null hypothesis that there is no significant difference between within- and between-subject Bray-Curtis dissimilarity for each of the 6 sample pairs. For all sample pairs, the within-subject similarity was greater than the between subject similarity. Despite the disparate anatomic sites, the 14–20 hour separation between oral samples, and the overnight fast prior to oral wash 2 sampling, subject-specific features of the microbiota drive significant within-subject sample similarity. It is also apparent, by reviewing the mean within-subject dissimilarities in column 2, that the two most similar sites on beta diversity analysis are oral wash 1-oral wash 2, followed closely by oral wash 1-sputum and oral wash 2-sputum. The sputum-nasal and both oral wash-nasal pairs are much less similar in β-diversity.

**Table 2:** Within- vs. between-subject sample similarity.

	Mean within-subject Bray-Curtis Dissimilarity	Mean between-subject Bray-Curtis Dissimilarity	p-value
Oral Wash 1 vs. Sputum	0.317	0.620	*p*<0.001
Oral Wash 1 vs. Nasal	0.958	0.967	*p*<0.001
Oral Wash 2 vs. Sputum	0.366	0.601	*p*<0.001
Oral Wash 2 vs. Nasal	0.960	0.968	*p* = 0.041
Oral Wash 1 vs. Oral Wash 2	0.302	0.582	*p*<0.001
Sputum vs. Nasal	0.945	0.961	*p* = 0.003

### Oral wash-sputum similarity is unaffected by an overnight fast

**Fig 7:**
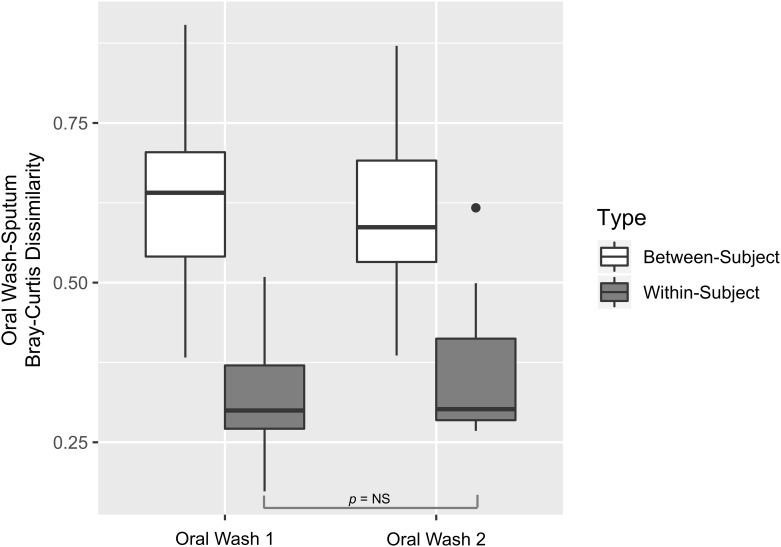
Oral wash-sputum similarity is unaffected by an overnight fast. To determine if oral wash 1 samples (obtained just prior to sputum sampling) are more similar to sputum samples than oral wash 2 samples (obtained the following morning after fasting) are similar to sputum samples, we calculated the Bray-Curtis dissimilarities between each subject’s sputum samples and oral wash 1 samples and each subject’s sputum samples and oral wash 2 samples. Within-subject oral wash-sputum dissimilarities are shown in gray while between-subject oral wash-sputum dissimilarities are shown in white. Oral wash 1 and oral wash 2 samples are presented separately. Inspection shows that there is greater within-subject than between-subject similarity, regardless of which oral wash sample is evaluated. Permutation testing demonstrated no significant difference between median oral wash 1-sputum dissimilarity and median oral wash 2-sputum within-patient dissimilarity (0.302 vs. 0.309, *p* = 0.99). Oral wash-sputum sample within-subject similarity is unaffected by obtaining oral wash samples 14–20 hours later after fasting, as opposed to oral wash sampling at the time of sputum collection.

To understand the similarity between the oral wash samples and the sputum samples, we compared the within- and between-subject Bray-Curtis dissimilarity for OW1-sputum and OW2-sputum separately. It is clear that there is significantly greater within-subject than between-subject similarity regardless of which oral wash sample is considered (Fig 7). To determine if OW1 samples (obtained just prior to sputum sampling) are more similar to sputum samples than OW2 samples (obtained the following morning after fasting) are similar to sputum samples, we calculated the Bray-Curtis dissimilarities between each subject’s sputum samples and OW1 samples and each subject’s sputum samples and OW2 samples. We tested the null hypothesis that there is no significant difference between median sputum-OW1 similarity and sputum-OW 2 Bray-Curtis dissimilarity within patients. The median within-subject Bray-Curtis dissimilarity between OW1 and sputum samples was 0.302 and the median dissimilarity between OW2 and sputum samples was 0.309. The *p*-value for this test was 0.99, consistent with the finding that both oral wash samples are very similar to the sputum samples.

### Age and severity of obstruction are associated with ASV abundance

To determine which taxa may be associated with relevant clinical characteristics, we investigated potential associations based on age, FEV1pp, and smoking status. We did not evaluate nasal samples or ICS use as there were too few samples available for comparison. In our analysis of oral samples, FEV1pp and smoking status were not significantly associated with any ASVs (all FDR > 0.10). Ten ASVs were associated with age (FDR < 0.10) in the oral wash samples; ASVs with FDR < 0.05 are listed in Table 3. Note that all listed ASVs increased in abundance with increasing age.

**Table 3:** Oral wash taxa associated with age.

Coefficient	FDR	Corresponding Genus
0.412	0.033	*Neisseria*
0.363	0.0081	*Veillonella*
0.323	0.0057	*Lautropia*
0.315	0.0094	*Streptococcus*
0.260	0.015	*Corynebacterium*

Among sputum samples, there were no ASVs significantly associated (FDR < 0.10) with smoking status. 51 ASVs in sputum were significantly associated with age (FDR < 0.10), and these 51 ASVs represented 25 unique genera. The 42 ASVs with FDR < 0.05 are listed in Table 4. Note that 6 genera are represented more than once among these 42 ASVs. *Prevotella* is represented by 8 unique ASVs while *Streptococcus* is represented by 5 unique ASVs. Interestingly, while most *Prevotella* and *Streptococcus* ASVs increase in sputum with increasing age, one ASV from each genus demonstrates the opposite association.

**Table 4:** Sputum taxa associated with age.

Coefficient	FDR	Corresponding Genus
0.391	< 0.0001	*Neisseria*
0.372	< 0.0001	*Prevotella*
0.334	0.0004	*Leptotrichia*
0.331	< 0.0001	*Streptococcus*
0.323	< 0.0001	NA
0.312	< 0.0001	*Lautropia*
0.292	< 0.0001	*Prevotella*
0.252	0.0073	*Veillonella*
0.242	0.0019	*Selenomonas*
0.233	0.0020	*Lactobacillus*
0.222	< 0.0001	*Oribacterium*
0.208	0.0017	*Prevotella*
0.202	< 0.0001	*Lactobacillus*
0.197	0.0084	*Bifidobacterium*
0.192	0.0283	*Fusobacterium*
0.179	0.0275	*Prevotella*
0.164	0.0001	*Prevotella*
0.160	0.0001	*Prevotella*
0.159	0.0275	*Stomatobaculum*
0.152	0.0330	*Megasphaera*
0.147	< 0.0001	*Cryptobacterium*
0.144	0.0134	*Lachnoanaerobaculum*
0.144	< 0.0001	*Corynebacterium*
0.136	0.0062	*Selenomonas*
0.135	0.0096	*Campylobacter*
0.133	0.0096	*Lachnoanaerobaculum*
0.121	0.0249	*Prevotella*
0.108	0.0017	*Alloscardovia*
0.102	0.0249	*Alloprevotella*
0.0880	0.0096	*Cardiobacterium*
0.0845	0.0275	*Streptococcus*
-0.0891	0.0018	*Streptococcus*
-0.0895	0.0010	*Leptotrichia*
-0.0918	0.0154	*Granulicatella*
-0.0994	0.0142	*Dialister*
-0.108	0.0275	*Oribacterium*
-0.128	0.0008	*Prevotella*
-0.129	< 0.0001	*Streptococcus*
-0.141	0.0438	*Streptococcus*
-0.152	< 0.0001	*Granulicatella*
-0.170	0.0096	*Fusobacterium*
-0.211	0.0126	*Fusobacterium*

Sixty-one ASVs in sputum were significantly associated with FEV1pp (FDR < 0.10), and these 61 ASVs represented 32 unique genera. The 52 ASVs FDR < 0.05 are listed in Table 5. Note that 10 genera are represented more than once among these 52 ASVs. *Prevotella*, *Capnocytophaga*, and *Fusobacterium* are each represented by 3 unique ASVs, while *Leptotrichia* is represented by 5 unique ASVs and *Streptococcus* is represented by 7 unique ASVs. While most *Prevotella* and *Streptococcus* ASVs decrease in sputum with increasing FEV1pp, one *Prevotella* ASV and two *Streptococcus* ASVs increased instead.

**Table 5:** Sputum taxa associated with lung obstruction severity.

Coefficient	FDR	Corresponding Genus
0.0645	0.0044	*Rothia*
0.0341	0.0209	*Stomatobaculum*
0.0337	0.0101	*Veillonella*
0.0323	0.0186	*Veillonella*
0.0234	0.0162	*Sneathia*
0.0233	0.0166	*Prevotella*
0.0231	0.0021	*Streptococcus*
0.0225	0.0284	*Butyrivibrio*
0.0202	0.0001	*Streptococcus*
0.0184	0.0466	*Alloprevotella*
0.0119	0.0271	NA
0.0107	0.0389	*Oribacterium*
-0.0121	0.0162	*Dialister*
-0.0127	0.0181	*Anaeroglobus*
-0.0145	0.0250	*Eikenella*
-0.0153	0.0162	*Leptotrichia*
-0.0180	0.0162	*Leptotrichia*
-0.0181	0.0437	*Lactobacillus*
-0.0184	0.0162	*Capnocytophaga*
-0.0187	0.0243	*Fusobacterium*
-0.0194	0.0117	*Alloscardovia*
-0.0197	0.0463	*Streptococcus*
-0.0204	0.0162	*Selenomonas*
-0.0216	0.0428	*Treponema*
-0.0216	0.0111	*Leptotrichia*
-0.0218	0.0463	*Lachnoanaerobaculum*
-0.0224	0.0345	*Streptococcus*
-0.0227	0.0166	*Treponema*
-0.0234	0.0463	*Campylobacter*
-0.0247	0.0271	*Peptostreptococcus*
-0.0252	0.0142	*Streptococcus*
-0.0280	0.0166	*Leptotrichia*
-0.0287	0.0162	*Prevotella*
-0.0289	0.0067	*Capnocytophaga*
-0.0290	0.0162	*Leptotrichia*
-0.0291	0.0243	*Mycoplasma*
-0.0309	0.0219	*Gemella*
-0.0317	< 0.0001	*Campylobacter*
-0.0326	0.0271	Ruminococcaceae_UCG-0
-0.0328	0.0166	*Fusobacterium*
-0.0332	0.0212	*Porphyromonas*
-0.0338	0.0304	*Capnocytophaga*
-0.0343	0.0000	NA
-0.0349	0.0002	*Anaeroglobus*
-0.0354	0.0089	*Actinomyces*
-0.0355	0.0111	*Streptococcus*
-0.0466	0.0111	*Prevotella*
-0.0482	0.0034	*Stomatobaculum*
-0.0490	0.0001	*Parvimonas*
-0.0570	0.0034	*Moraxella*
-0.0749	< 0.0001	*Fusobacterium*
-0.0768	< 0.0001	*Streptococcus*

### Frequent exacerbator phenotype is associated with decreased alpha diversity independent of ICS use

Our ability to evaluate the effects of ICS use is limited because only 2 subjects in our study were using ICSs. To understand if ICS use is correlated with lung microbiota characteristics, we combined this dataset of mostly ICS non-users with data from our previous study of exacerbation phenotype, which included many ICS users (study approved by the Minneapolis VA Medical Center Institutional Review Board #4541-B) [30]. Although subjects from both these studies were similar with respect to age and gender, they differed with respect to FEV1pp, ICS use, and number of exacerbations in the last year (Table 6).

**Table 6:** Subject characteristics by study.

Study	Present Study	Exacerbation Phenotype Study [30]	
Phenotype	Infrequent Exacerbators	Infrequent Exacerbators	Frequent Exacerbators
n	15	9	10
Age, mean (sd)	65.20 (4.51)	68.36 (5.45)	70.82 (7.12)
Gender, Male (%)	14 (93.33)	9 (100)	10 (100)
BMI, mean (sd)	27.65 (5.98)	29.25 (6.01)	26.09 (6.85)
Race, Caucasian/White (%)	14 (93.3)	9 (100)	10 (90.9)
COPD Severity, n (%)^a^			
Mild [FEV1pp ≥ 80%]	5 (33.3)^a^	0 (0.00)	0 (0)
Moderate [FEV1pp = 50–79%]	7 (46.6)^a^	4 (44.4)	4 (40.0)
Severe [FEV1pp 30–49%]	2 (13.3)^a^	3 (33.3)	2 (20.0)
Very Severe [FEV1pp <30%]	0 (0.00)^a^	2 (22.2)	4 (40.0)
ICS Use, Yes (%)	2 (13.3)	6 (66.7)	7 (70.0)
Pack-years of smoking, mean (sd)	49.13 (19.58)	61.18 (31.94)	69.55 (48.57)
Current tobacco use, Yes (%)	3 (20.0)	5 (55.6)	3 (27.2)
Diabetes, Yes (%)	1 (6.7)	2 (22.2)	3 (30.0)
Edentulous, Yes (%)	3 (20.0)	Not assessed	Not assessed
Current alcohol use, Yes (%)	9 (60.0)	7 (77.8)	9 (81.8)
Oral steroid or antibiotic use in last 1 month, Yes (%)	0 (0)	0 (0)	0 (0)
COPD exacerbations in the last 12 months, mean (sd)	0 (0)	0 (0)	2.9 (1.1)
Lung cancer, Yes (%)	14 (93.3)	0 (0)	0 (0)

^a^One subject in the present study did not have COPD by pulmonary function criteria

When both studies are combined, sputum of ICS users is less diverse than sputum from non-ICS users by both Shannon (*p* = 0.0008) and Simpson indices (*p* = 0.008). To evaluate the relationship between individual clinical predictors and Shannon diversity, we used linear mixed models. When exacerbation phenotype was included in the model, site (oral vs. sputum) was significant (*p* = 0.008), while exacerbation phenotype was not significant. Individual inclusion of ICS use, age, and FEV1pp with exacerbation phenotype resulted in significant effects for phenotype in each model, but non-significant effects for ICS use, age and FEV1pp. Because the effects of ICS use may differ based upon exacerbation phenotype or site sampled (oral vs. sputum), models individually examining ICS use:phenotype and ICS use:site interactions were performed. These models demonstrated that when phenotype, ICS use, and phenotype:ICS use interactions were included, all factors were non-significant. When phenotype, ICS use, site, and ICS use:site interactions were included, only phenotype was significant (*p* = 0.024). These results suggest that exacerbation phenotype, rather than ICS use, is more closely associated with changes in α-diversity.

## Discussion

Our study is the first to specifically target relevant clinical characteristics such as age, ICS use, current tobacco use, presence of teeth, and degree of lung obstruction with the upper airway and sputum microbiota. Here we have shown potential associations between age and both oral wash and sputum taxa abundance; obstruction severity and sputum taxa abundance; edentulous state and beta diversity; and ICS use and beta diversity. In addition, overnight fasting may be associated with increased bacterial burden in the oral microbiota. Furthermore, one subject’s samples were consistent outliers on both alpha and beta diversity analyses, suggesting that potential associations between these microbiota characteristics and gender or tumor burden should be investigated.

Although it may be expected that edentulous subjects would harbor fewer oral bacteria, our data obtained both mid-day and after an overnight fast did not support this conclusion. Surprisingly, oral bacterial biomass increased following an overnight fast when compared to mid-day samples. Daytime oral activities such as toothbrushing, swallowing, eating, and drinking may decrease the oral bacterial burden compared to samples obtained after an overnight fast.

Our studies using alpha diversity demonstrate lower nasal diversity compared to oral wash and sputum samples. Comparison of the Shannon diversity oral wash and sputum values and the Simpson diversity oral wash and sputum values obtained in this study (3.19, 3.31, 0.91, and 0.92, respectively) with our previous work on COPD frequent and infrequent exacerbators shows that our mean Shannon and Simpson values in this study are on par with the values obtained for infrequent exacerbators in our last study [30]. In contrast, the alpha diversity of the frequent exacerbators’ oral washes and sputa was significantly lower than the alpha diversity values obtained here, particularly among the sputum samples. Consistent with these observations about alpha diversity, subjects in this study all reported no prior COPD exacerbations. We also did not identify any associations between alpha diversity and clinical factors (age, ICS use, obstruction severity, current tobacco use, or edentulous state).

Our clustering, beta diversity, and PERMANOVA analyses demonstrate a high degree of similarity between subjects’ paired oral wash samples, which were also very similar to their sputum samples. In contrast, nasal samples separated from the oral wash and sputum samples in multiple analyses. PERMANOVA analyses demonstrated clustering based on ICS use and edentulous state, but not related to obstruction severity or current tobacco use.

Our analysis of taxa abundance by site and clinical factors demonstrated significant taxa shifts in oral samples associated with increased age. Significant taxa shifts in sputum samples were also noted in association with age or severity of obstruction. Most of the taxa identified in these analyses were common oral or sputum taxa (*Actinomyces*, *Fusobacterium*, *Moraxella*, *Prevotella*, *Rothia*, *Streptococcus*, *Veillonella*). In instances where associations between multiple ASVs from the same genus (such as *Streptococcus* or *Prevotella*) were identified, there was not always uniformity in the direction of the change in abundance. For instance, most sputum ASVs mapping to *Streptococcus* and *Prevotella* increased in association with increasing age and decreased in association with less severe obstruction. However, one or two ASVs mapping to these two genera demonstrated the opposite correlations. This illustrates both the breadth and complexity of the Streptococci as well as the role that speciation or whole-genome sequencing may play in understanding the complex relationship between the microbiota and the immune system.

Because our data set did not contain sufficient numbers of ICS users to thoroughly evaluate microbiota associations with ICS use, we combined the current study with the dataset from an exacerbation phenotype study, in which ~70% of subjects were ICS users [30]. Caution must be taken in interpreting these analyses, as ICS use was closely tied to study population and, to a lesser extent, exacerbation phenotype. Furthermore, the patient populations demonstrated differences in lung function, as shown in Table 6. Our combined analysis of both oral wash and sputum alpha diversity took both patient effects and exacerbation phenotype effects into account. The effect of exacerbation frequency was significant when ICS use was taken into account, but ICS use was not a significant predictor of alpha diversity when phenotype, anatomic site, or their interactions with ICS use were considered. Exacerbation phenotype, rather than ICS use, appears to be associated with changes in alpha diversity. These findings should be interpreted cautiously, however, as other clinical factors that are associated with exacerbation phenotype and ICS use (such as age or obstruction severity) could be responsible for the decreased alpha diversity instead.

Additionally, samples from one of our subjects were outliers in both alpha and beta diversity analyses. Due to these striking shifts, we first ensured that the beta diversity clustering and decreased alpha diversity were not due to DNA extraction batch effects. Review of laboratory records showed that these 4 samples were extracted in 3 different batches on 3 different days. None of the other subjects’ samples processed alongside these 4 samples exhibited similar clustering or very low alpha diversity. All samples were sequenced in the same MiSeq lane. Once we ensured that these findings were due to the biological nature of the samples, rather processing or sequencing effects, we considered possible clinical associations. This subject was not only the only woman in our dataset, but also the only subject who was diagnosed with multiple lung tumors simultaneously (two independent adenocarcinomas and a squamous cell carcinoma). Future studies should evaluate the effects of gender and lung tumor burden on the lung and upper airway microbiota.

Our study had several strengths. We were able to assess the associations of multiple clinical factors on both the upper airway and sputum microbiota simultaneously. We showed that the oral microbiota is sufficiently stable over ~24 hours and following an overnight fast to maintain significant within-subject similarity. By combining two datasets, we were able to evaluate potential associations between ICS use and the sputum microbiota—of significant clinical interest given the widespread use of ICS to treat COPD and recent changes to the GOLD guidelines on the use of ICS in this patient population. Our study also had several weaknesses. Our patients’ lung tumors may have altered their oral and lung microbiota, restricting the generalizability of our findings. We were likely underpowered to detect some associations. One of our subjects did not meet lung function criteria for COPD. Despite analyzing FEV1pp as a continuous variable, inclusion of this subject with a FEV1/FVC ratio ≥ 0.70 may have impacted our conclusions. Furthermore, any correlations identified in this study should not be assumed to play a causative role; interventional randomized studies are necessary to address causation. In particular, our analysis of ICS use and exacerbation phenotype should be interpreted cautiously given the number of other colinear predictors (study population, age, obstruction severity, etc.) that may be driving the association observed here. Nevertheless, our data support the observation that several clinical factors in addition to disease severity are associated with oral and sputum microbiota changes.

## Conclusions

Among the upper airway microbiota of COPD subjects, anatomic site was associated with bacterial biomass, Shannon diversity, and beta diversity. ICS use and edentulous state were both associated with beta diversity. Age was associated with taxa relative abundance in oral and sputum samples, while FEV1pp was associated with taxa relative abundance in sputum samples only.
